# Supplementary figures and images for: Active suppression of a leaf meristem orchestrates determinate leaf growth
Source: eLife. 2016 Oct 6;5:e15023. doi: 10.7554/eLife.15023 (PMC5096885; doi:10.7554/eLife.15023)

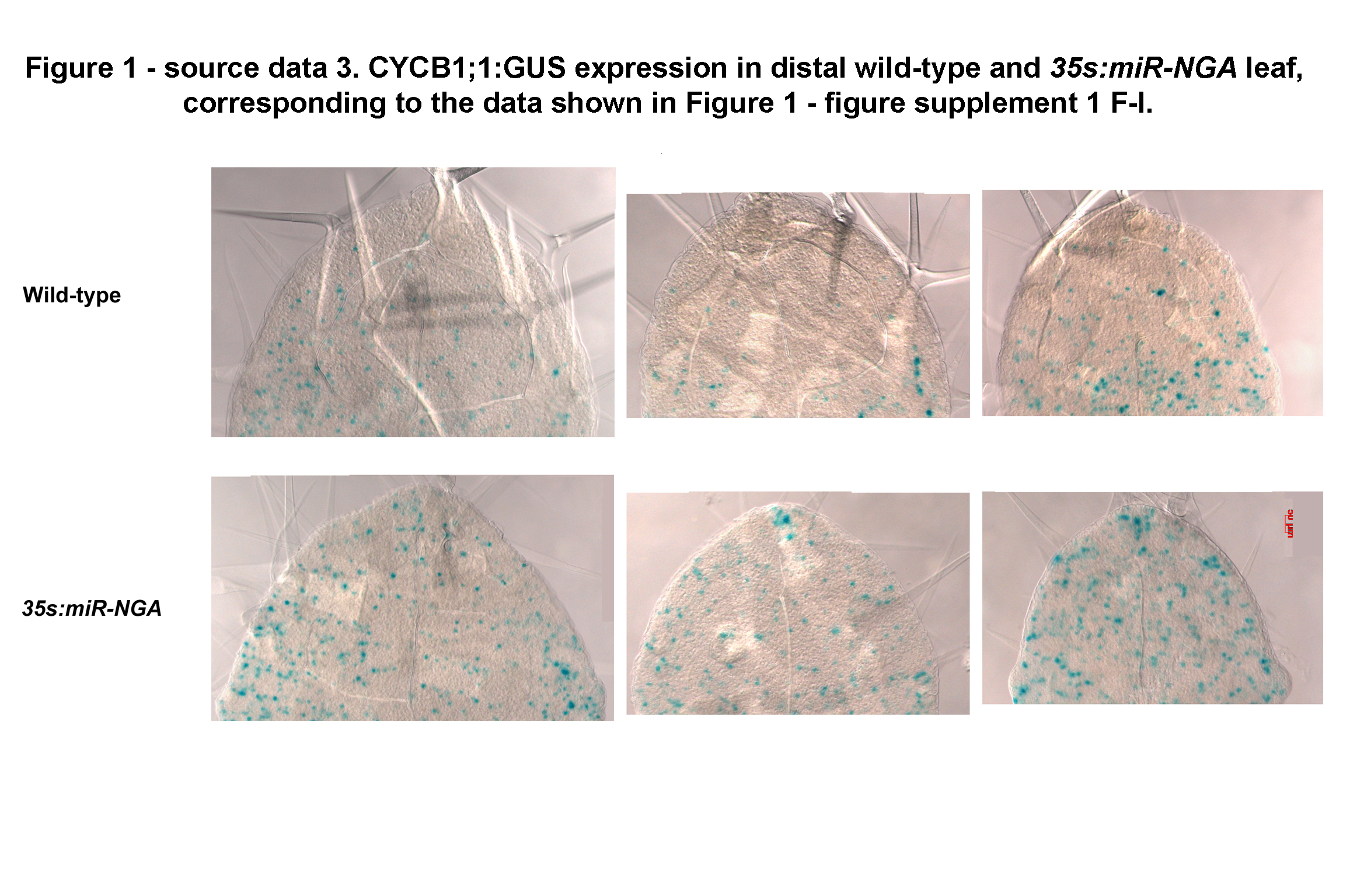

Supplement: Figure 1—source data 3. — DOI: http://dx.doi.org/10.7554/eLife.15023.005 [file elife-15023-fig1-data3.jpg]
