# Supplementary material for: Active suppression of a leaf meristem orchestrates determinate leaf growth
Source: eLife. 2016 Oct 6;5:e15023. doi: 10.7554/eLife.15023 (PMC5096885; doi:10.7554/eLife.15023)
Supplement: Supplementary file 1. — DOI: http://dx.doi.org/10.7554/eLife.15023.030 [file elife-15023-supp1.docx]

**Supplementary file 1.** **Primers used for PCR-mediated cloning.**

| Cloning primers | Sequence 5’ to 3’ |
| --- | --- |
| *PRS* promoter F | TTCTGCAGGCGAGTCGTACTACGAAACCAAACAAT |
| *PRS* promoter R | AGGGTACCTTCTCCGTTCAGACAAATATTGATGCT |
| *WOX1* promoter 5’ F | CTGCAGAACTTTCGTAGCAATCTCTGTTTCTCTCTTTCAAAACCGG |
| *WOX1* promoter 5’ R | CTCGAGTTTGGTGTGTACTTAATTTATATGTATGTTTATAGAGGAG |
| *WOX1* promoter 3’ F | GGATCCTCTAGAACCTAATTGAAGTCTTTCAAGAAAAACAGCTTTTTG |
| *WOX1* promoter 3’ R | ACTAGTGCAAATCGAAATCTACTACTTCTATGTTTTCTTCTTTC |
| *TCP4* promoter *5’ NotI* F | GCGGCCGCGAAGTGTGAGAGTGTGATAGGACATCATGTCCC |
| *TCP4* promoter *5’ XhoI* R | CTCGAGGGTAGAGCATATTCGTCGAGACGGCGGCG |
| *TCP4* promoter *3’* *miR319* target F | GGATCCTGAGAGGGGTCCCCTTCAGTCCAGCAATCGAAACTAATCCTCTAAGTTTCAGG |
| *TCP4* promoter *3’ NotI* R | GCGGCCGCTTCACATGTTGATGTTGGTGAGAGAGAGC |
| *TCP3* promoter 5’ *NotI* F | GCGGCCGCGGAGGACTTGCATAGGTAGAGTTGATGTTAAATG |
| *TCP3* promoter 5’ *XhoI/PstI* R | CTGCAGCTCGAGTGATGAAGCATATTGTGGAAATTCAAGGTCGTTGATG |
| *TCP3* promoter 3’ *mir319* target F | CCCGGGGAGGGGTCCCCTTCAGTCCATACAAGTTCCAAAAACACATAAGATTGTGCAACACAACAGG |
| *TCP3* promoter 3’ *NotI* R | GCGGCCGCTTGAGTCTTTATTATTTTCGATAGAATTCTTATTTAC |
